# Supplementary figures and images for: Have female twisted-wing parasites (Insecta: Strepsiptera) evolved tolerance traits as response to traumatic penetration?
Source: PeerJ. 2022 Aug 16;10:e13655. doi: 10.7717/peerj.13655 (PMC9390352; doi:10.7717/peerj.13655)

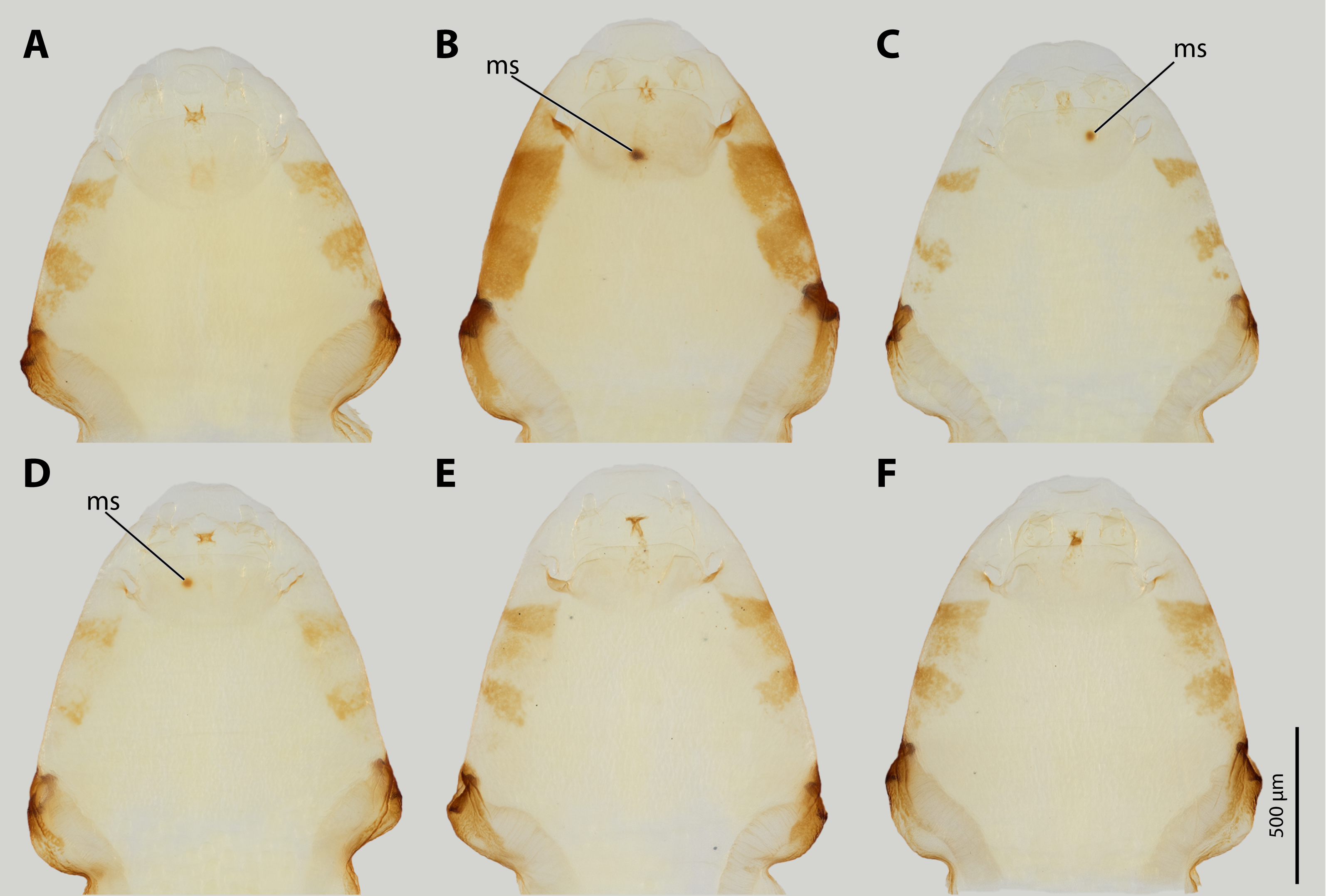

Supplement: Supplemental Information 1 — (A, E, F) Female Stylops ovinae without mating sign after allospecific mating attempts. (B, C, D) Female Stylops ovinae with injurys from allospecific mating attempts. Abbreviation: ms – mating sign. [file peerj-10-13655-s001.png]

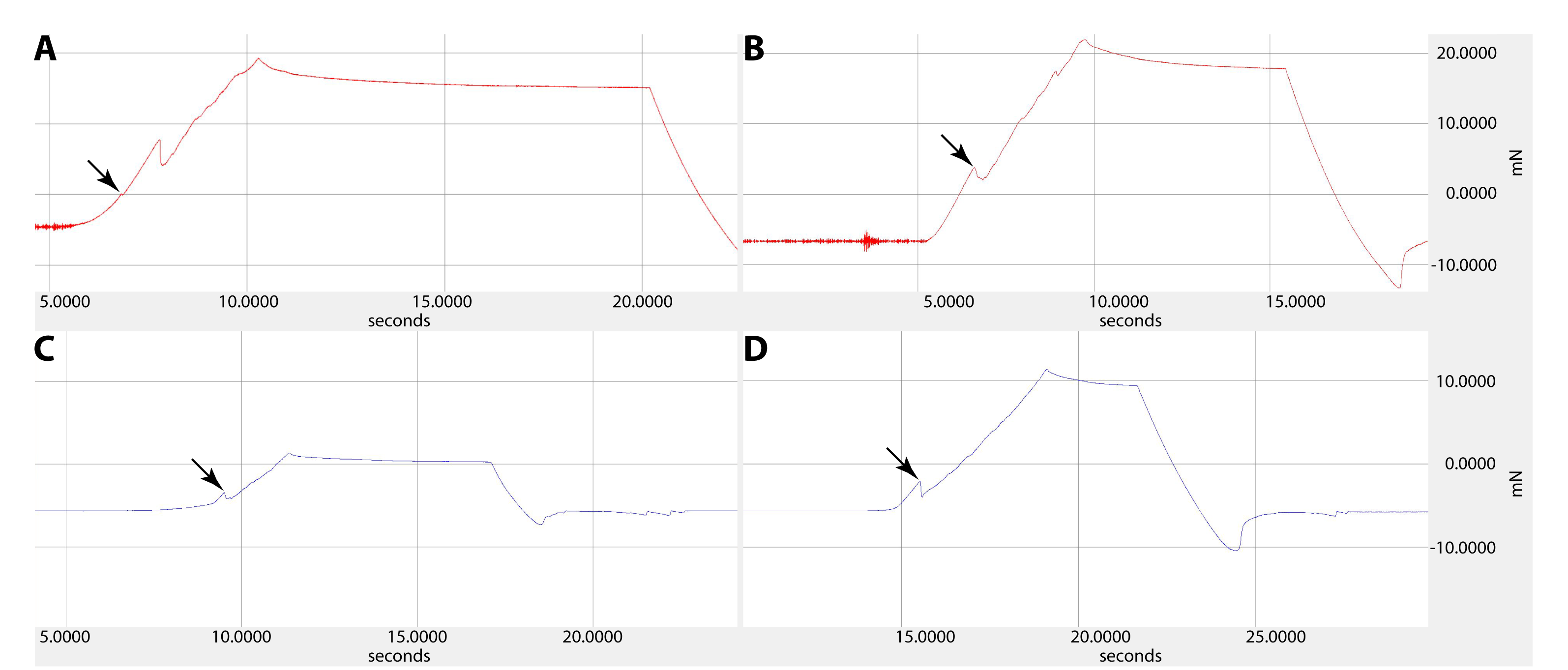

Supplement: Supplemental Information 2 — (A) Control site of Stylops ovinae. (B) Wounding site of Stylops ovinae (C) Control site of Xenos vesparum. (D) Wounding site of Xenos vesparum. Star indicates penetration of either control or wounding site. [file peerj-10-13655-s002.png]

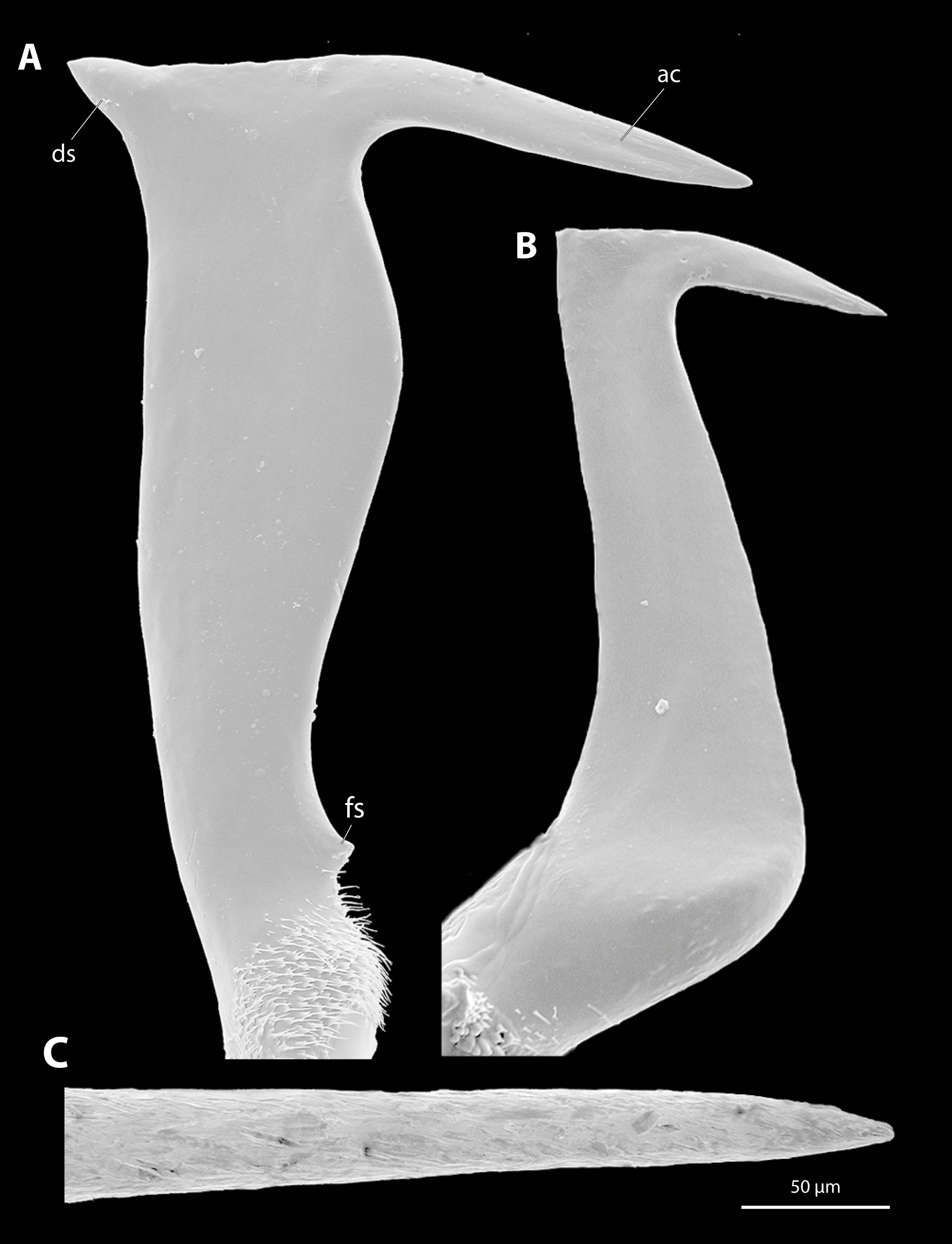

Supplement: Supplemental Information 3 — (A) Penis of Stylops ovinae, lateral view. (B) Penis of Xenos vesparum, lateral view. (C) Tip of the microneedle. Abbreviations: ac – acumen, ds – dorsal spine, fs – frontal spine. [file peerj-10-13655-s003.png]
